# Astragaloside IV-Loaded Polydopamine/Zeolitic Imidazolate Framework-8 Nanoparticles Embedded in Conductive Decellularized Extracellular Matrix-Modified Hydrogels for Wound Healing

**DOI:** 10.3390/pharmaceutics18060726

**Published:** 2026-06-12

**Authors:** Xingjian Liu, Wei Zhang, Guanyong Deng, Haozhe Yu, Shilin Tian, Jiahui Liu, Wenzeng Hu, Tianyu Pan, Lihong Fan

**Affiliations:** School of Chemistry, Chemical Engineering and Life Sciences, Wuhan University of Technology, 122 Luoshi Road, Wuhan 430070, China; 349070@whut.edu.cn (X.L.); 349065@whut.edu.cn (G.D.);

**Keywords:** conductive hydrogel, carboxymethyl chitosan, decellularized extracellular matrix, PDA/ZIF-8, astragaloside IV, wound healing

## Abstract

**Background:** Conventional and refractory wounds frequently remain in a prolonged inflammatory phase associated with excessive reactive oxygen species (ROS) accumulation and disruption of endogenous electrical cues. **Methods:** A multifunctional nanocomposite hydrogel was fabricated via an amidation condensation reaction, utilizing 3-amino-4-methoxybenzoic acid (AMB)-modified carboxymethyl chitosan (PAMB-CMCS) and decellularized extracellular matrix (dECM) as macromolecular networks, integrated with Astragaloside IV-Loaded Polydopamine/Zeolitic Imidazolate Framework-8 (AS@PDA/ZIF-8) nanoparticles. **Results:** The hydrogel provided a biomechanically supportive scaffold with compressive strength of 27.24 ± 1.9 kPa and breaking strength of 28.2 ± 2.8 kPa and exhibited electrical conductivity of 29.84 mS/cm, ROS-scavenging activity, and near-infrared (NIR)-responsive photothermal behavior reaching 62.55 °C. The integrated PDA@ZIF-8 nanoplatform further contributed to antibacterial performance and localized AS release, thereby improving the wound microenvironment and accelerating full-thickness cutaneous defect repair. **Conclusions:** This macromolecule-based composite hydrogel offers a promising therapeutic strategy for complex wound management.

## 1. Introduction

As the largest organ of the human body, skin serves as a vital barrier for maintaining internal homeostasis and protecting against external pathogens and harmful stimuli. Wound healing is a complex and dynamic process involving inflammation regulation, cell proliferation and migration, angiogenesis, and extracellular matrix (ECM) remodeling [1]. Recent advances in dynamic tissue engineering further indicate that tissue regeneration is not a static repair event, but a continuously evolving process in which cells, ECM deposition, biochemical signals, and the surrounding physical microenvironment interact throughout the regenerative window. However, extensive full-thickness wounds often suffer from delayed healing, bacterial infection, excessive inflammation, and scar formation, which impair both functional and aesthetic recovery [2,3,4,5]. Conventional wound dressings mainly provide passive physical protection and are generally insufficient to actively regulate the complex regenerative microenvironment. Therefore, bioactive wound dressings that combine ECM-like structural support, biological regulation, electrical conductivity, and controlled drug delivery have attracted increasing attention in skin tissue repair [6]. In this context, multifunctional hydrogel dressings capable of simultaneously providing structural support, localized therapeutic delivery, and microenvironmental modulation are particularly promising for full-thickness wound repair [7,8,9].

Hydrogels are widely used as wound dressings because of their high water content, porous three-dimensional networks, and favorable tissue compatibility. Hydrogel networks can partially mimic the native ECM architecture and can also function as reservoirs for sustained delivery of bioactive molecules, thereby enabling active regulation of local tissue responses rather than merely serving as passive coverings. Carboxymethyl chitosan (CMCS), a water-soluble chitosan derivative, possesses biodegradability, hydrophilicity, and good processability, making it a suitable polymer for constructing wound-healing hydrogels [10,11]. Nevertheless, pristine CMCS hydrogels usually exhibit limited mechanical strength and lack sufficient functional cues for regulating wound repair. In particular, because endogenous electrical signals are involved in skin repair, electrically inert hydrogels may not adequately support bioelectrical communication associated with cell migration, proliferation, and angiogenesis [12,13,14]. Moreover, recent studies on mechanically adaptive hydrogels suggest that hydrogel network organization and dynamic remodeling can provide important biophysical cues for cell alignment and tissue-like organization, highlighting the importance of designing hydrogels with both biochemical and physical regulatory functions.

Conductive hydrogel systems provide a potential strategy to address this limitation. Previous studies have shown that conductive biomaterials can support wound healing by providing electroactive microenvironments and promoting wound closure-related cellular responses [15,16,17]. In addition to electrical conductivity, the long-term stability and uniform distribution of electroactive components within hydrogels are critical for maintaining functional performance under repeated deformation at wound sites. However, many conductive hydrogels are prepared by physically blending conductive polymers or carbon-based nanomaterials, which may lead to aggregation, uneven distribution, or potential leaching [18,19]. In this study, AMB was grafted onto the CMCS backbone to construct a conductive PAMB-CMCS hydrogel matrix. This chemical modification was expected to improve the distribution stability of electroactive components within the hydrogel network. To further enhance the biological microenvironment, dECM derived from porcine Achilles tendon was incorporated. dECM retains native ECM components such as collagen and elastin, which may provide cell-adhesive sites and biological support for tissue regeneration [20,21,22]. The incorporation of dECM is also consistent with recent bio-adaptive hydrogel concepts, in which ECM-like matrices are used to support cell migration, matrix remodeling, and tissue maturation during regeneration.

In addition to structural and electrical support, regulation of inflammation and oxidative stress is important for wound repair. AS is the main active ingredient of Astragali Radix and has been reported to have anti-inflammatory and antioxidant activities, including the regulation of TNF-α, IL-6, and NF-κB related pathways [23,24,25]. However, its direct application is limited by poor stability, rapid degradation, and low bioavailability. ZIF-8 is a porous nanocarrier with pH-responsive degradation behavior, while PDA provides adhesive properties, photothermal responsiveness, and abundant surface functional groups [26,27,28]. Hydrogel-based local delivery platforms have been shown to prolong the retention and release of therapeutic agents in situ, which provides a useful design rationale for integrating drug-loaded nanocarriers into a wound dressing matrix. Therefore, PDA-coated ZIF-8 nanoparticles were used to load AS, forming AS@PDA/ZIF-8 (APZ8) nanocarriers for incorporation into the hydrogel system.

**Figure 1:**
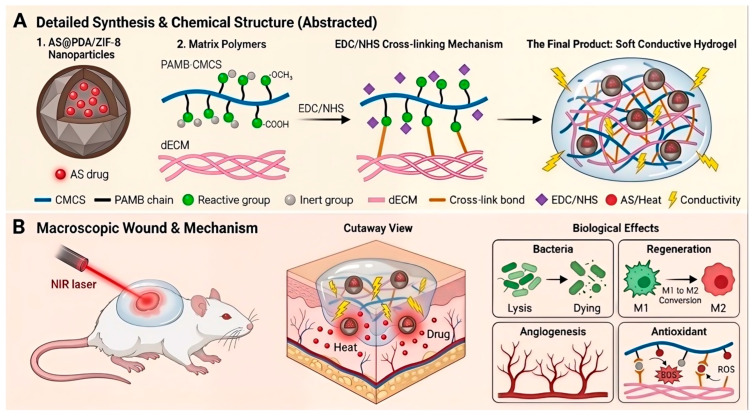
Construction of APZ8H hydrogel. (**A**) Schematic diagram of the hydrogel preparation mechanism. (**B**) The mechanism diagram of the hydrogel applied to wound healing in rats.

Herein, a multifunctional nanocomposite hydrogel, APZ8H, was developed by integrating AS-loaded PDA@ZIF-8 nanoparticles into a conductive PAMB-CMCS/dECM hydrogel network. The PAMB-CMCS matrix was constructed through AMB modification of CMCS, followed by 1-ethyl-3-(3-dimethylaminopropyl)carbodiimide hydrochloride/N-hydroxysuccinimide (EDC/NHS)-mediated crosslinking with dECM. This design integrates an ECM-like hydrogel scaffold, a chemically stabilized conductive polymer network, and a nanocarrier-mediated sustained drug-delivery module to construct a dynamic wound-regulating microenvironment, the mechanism diagram is shown in Figure 1. The obtained hydrogel was systematically evaluated in terms of morphology, chemical structure, swelling behavior, mechanical properties, degradation, conductivity, antioxidant activity, antibacterial performance, and cytocompatibility. Furthermore, its wound repair performance was investigated using a full-thickness skin defect model, together with hematoxylin and eosin (H&E) and Masson staining to assess tissue regeneration and collagen deposition.

## 2. Materials and Methods

### 2.1. Materials

Dopamine (DA, 98%) and 2-methylimidazole (2-MIM, 98%) were purchased from Shanghai Aladdin Biochemical Technology Co., Ltd. (Shanghai, China). Zinc nitrate hexahydrate (Zn(NO_3_)_2_·6H_2_O, AR). Methanol (AR), carboxymethyl chitosan (CMCS), sodium hydroxide (NaOH), ammonium hydroxide (NH_4_OH), acetic acid (CH_3_COOH, HAc), 3-amino-4-methoxybenzoic acid (AMB), and hydrochloric acid (HCl) were purchased from Sinopharm Chemical Reagent Co., Ltd. (Shanghai, China). Fresh porcine Achilles tendon was obtained from Wuchang Slaughterhouse (Wuhan, China). Phosphate-buffered saline (PBS), sodium dodecyl sulfate (SDS, CH_3_(CH_2_)_11_OSO_3_Na), N-hydroxysuccinimide (NHS), Astragaloside IV (AS), 1-ethyl-3-(3-dimethylaminopropyl)carbodiimide (EDC) and Pepsin (Pep) were purchased from Shanghai Macklin Biochemical Technology Co., Ltd. (Shanghai, China). Triton X-100 was obtained from Promega Corporation (Madison, WI, USA). All raw materials were used directly without further purification.

### 2.2. Preparation of ZIF-8 NPs

Briefly, Zn(NO_3_)_2_·6H_2_O (1.465 g) was dissolved in deionized water (20 mL). Subsequently, 2-methylimidazole (3.245 g) was completely dissolved in an ammonium hydroxide solution (20 mL). The aforementioned zinc nitrate solution was then added to the 2-methylimidazole solution under continuous stirring, initially forming a gray reaction mixture. After 1 h of constant stirring, the mixture evolved into a milky-white suspension, indicating the successful formation of ZIF-8 nanoparticles. The resulting precipitates were collected by means of centrifugation at 8000 rpm for 10 min. Finally, the product was washed thrice with deionized water and methanol to remove unreacted precursors and dried under vacuum at 60 °C for 12 h to yield the pristine ZIF-8 as a white powder.

### 2.3. Preparations of PDA/ZIF-8

The prepared ZIF-8 50 mg was dispersed in 50 mL Tris buffer, pH = 8.5, adjusted to provide conditions for oxidative self-polymerization of dopamine, and then dopamine hydrochloride was added to the above dispersion and stirred at room temperature for 24 h. The resulting nanoparticles were collected via centrifugation at 10,000 rpm and washed thrice with deionized water and absolute ethanol. The final product was dried in a vacuum oven at 40 °C for 2 h.

### 2.4. Preparations of AS@PDA/ZIF-8

The 1 mg/mL ZIF-8 solution was mixed with 1 mg/mL AS solution and stirred at room temperature for 2 h. Next, 0.5 mg/mL of PDA solution was added and the pH was adjusted to 8.5. The reaction was carried out at 25 °C for 1 h and centrifuged at 10,000 rmp for 10 min; the supernatant was washed three times with anhydrous ethanol and dried at 40 °C in vacuum.

### 2.5. Preparation of 0.8% dECM

The method for dECM preparation followed a previously established protocol. First, the tissue was excised using a scalpel to remove surface fat and fascia and then minced. For surfactant treatment, the minced porcine Achilles tendon was immersed in deionized water containing 2% Triton X-100 and 1% sodium dodecyl sulfate (SDS) at a mass ratio of 1:50 (tendon to solution). The mixture was stirred at room temperature with a magnetic stirrer for 20–24 h. The tendon tissue from the previous step was thoroughly washed with deionized water to remove all residual Triton X-100 and SDS. It was then frozen in liquid nitrogen for 10–15 min. Subsequently, the tissue was thawed in deionized water at 37 °C until completely thawed (approximately 30 min). This freeze–thaw cycle was repeated five times. The thawed tendon tissue was then added to a 1% acetic acid solution (using the same volume ratio as in the second step) and continuously stirred with a magnetic stirrer at room temperature for 30–36 h. The acid-treated tissue was washed thoroughly with deionized water until the pH exceeded 5 to remove all acid. After draining and weighing, the tissue was transferred to a homogenizer with an ice-water mixture and homogenized into a smooth paste (ensuring no granular texture remained). The solid content of this ECM slurry was then measured. The ECM slurry was diluted to a solid content of 0.8%. A 100 g aliquot of this sample was taken, and 0.08 g of pepsin was added. The mixture was stirred with a magnetic stirrer at 37 °C for 20–24 h. Following the digestion step, 0.3 mL of Triton X-100 was added. The sample was homogenized in a beaker using a homogenizer at 4000 rpm for 15 min. The resulting foam was poured into a mold and thoroughly frozen in a freezer. After complete lyophilization for 48–54 h, the artificial dermal sponge was obtained.

### 2.6. Synthesis of PAMB-CMCS Copolymer

Briefly, 10 g of CMCS powder was dissolved in 25 mL of deionized water at 40 °C to yield a 40% (*w*/*v*) polymer solution. 1.5 g of AMB was added to the solution, and the mixture was stirred at 600 rpm for 36 h. Subsequently, 273 mg of ammonium persulfate (APS) was introduced, and the reaction temperature was elevated to 50 °C for 24 h. Upon completion, the synthesized PAMB-CMCS polymer was dialyzed against deionized water at 37 °C for 3 days, with the dialysate refreshed every 12 h. Finally, the PAMB-CMCS product was lyophilized for 48 h and stored in a desiccator at room temperature. To fabricate the composite hydrogels, a 0.8 wt% dECM aqueous solution and a 5 wt% PAMB-CMCS aqueous solution were separately prepared. These solutions were homogeneously mixed at PAMB-CMCS/dECM mass ratios of 5:0, 4:1, 3:2, and 2:3, followed by incubation at 37 °C for 18 h. Thereafter, 20 mg of EDC/NHS were added. The mixtures were further incubated at 37 °C for 3 h to facilitate chemical crosslinking, yielding the final PAMB-CMCS/dECM hydrogels. The resulting hydrogels were designated as P_5_D_0_, P_4_D_1_, P_3_D_2_, and P_2_D_3_, respectively, corresponding to their mass ratios.

### 2.7. Synthesis of PZ8H and APZ8H

Initially, a 0.5 wt% PAMB-CMCS aqueous solution and a 0.8 wt% dECM solution were prepared. Subsequently, the two solutions were homogeneously mixed at a mass ratio of 4:1 (PAMB-CMCS to dECM) and incubated at 37 °C under continuous shaking at 200 rpm for 24 h. Following the addition of 20 mg of EDC and NHS to initiate crosslinking, the resulting hydrogel was designated as P_4_D_1_. To prepare the nanocomposite hydrogels, 20 mg of either PDA/ZIF-8 or AS@PDA/ZIF-8 nanoparticles were introduced into the respective mixtures and sonicated for 30 min to ensure uniform dispersion. Finally, 20 mg of EDC and NHS were added to each respective mixture, followed by a 1 h reaction at 37 °C to yield the fully crosslinked PZ8H and APZ8H composite hydrogels.

### 2.8. Characterization

#### 2.8.1. Characterization of Nanoparticles

The prepared above NPs were respectively characterized by using X-ray diffraction (XRD, MiniFlex 600, Rigaku Corporation, Tokyo, Japan) and a Fourier transform infrared spectrometer (FTIR, IRAffinity-1S, Shimadzu Corporation, Kyoto, Japan). The drug loading and release of nanoparticles were analyzed via high-performance liquid chromatography (HPLC) (1260, Agilent, Waldbronn, Germany) with a ShimNex CS C18 (4.6 mm × 150 mm, 5 μm) column equipped with an evaporative light detector (3300, Alltech Associates, Deerfield, IL, USA). The morphology of NPs was observed via a transmission electron microscope (TEM, Hitachi, Tokyo, Japan) coupled with energy-dispersive X-ray spectroscopy (EDS). The Zeta potential and nanomaterial size in DI water were measured using a Nano-ZS (Malvern Panalytical, Shanghai, China). X-ray photoelectron spectroscopy (XPS) was performed on an ESCALAB 250Xi (Thermo Fisher, Waltham, MA, USA). The specific surface area and drug loading rate of nanoparticles were tested using the Brunauer–Emmett–Teller method (BET, Micromeritics, Norcross, GA, USA).

#### 2.8.2. Characterization of dECM

A small number of dECM sponge samples were weighed and placed in an oven at 37 °C for 48 h to remove moisture. The control group was collagen (Col). The samples were scanned using a Fourier transform infrared spectrometer with a scanning range of 400–4000 cm^−1^ and then the FT-IR of dECM and Col was compared and qualitatively analyzed. The 0.8 wt% dECM sponge was cut into highly consistent squares, and the conductive adhesive was used to adhere to the carrier stage. Then, the surface of the sample was sprayed with gold, and finally, the structure of the surface of the material was observed via SEM. The fresh porcine Achilles tendon tissue and the decellularized porcine Achilles tendon tissue were cut into slices with a thickness of about 3 mm. After that, the slices were fixed with formaldehyde solution and immersed in different concentrations of ethanol solution in turn to dehydrate. The dehydrated slices were embedded in paraffin and sliced. Finally, the sheet was dewaxed with xylene. The dewaxed slices were rehydrated and stained with HE for 10 min. The stained slices were immersed in hydrochloric acid–alcohol differentiation solution for 20 s and then washed with flowing deionized water for 5 min. After that, the slices were dyed with eosin solution for 3 min, followed by washing, dehydration and sealing. Finally, the dyed tissues were observed using a microscope.

#### 2.8.3. Characterization of PAMB-CMCS/dECM Hydrogels and AS@PDA/ZIF-8 Composite Hydrogel

The PAMB-CMCS/dECM hydrogel samples were dried to a constant weight in a forced-air oven and subsequently ground into a fine powder. This powder was homogeneously mixed with fully desiccated potassium bromide (KBr) powder at a mass ratio of 1:30 and pressed into pellets. The absorption spectra were recorded in the range of 400–4000 cm^−1^ using FTIR. To examine their internal morphology, the hydrogel samples were cryo-fractured using liquid nitrogen to expose a clean cross-section. Following lyophilization for 48 h, the fractured surfaces were sputter-coated with gold. The internal porous architectures of the hydrogels were subsequently imaged using an SEM.

#### 2.8.4. Performance Test of PAMB-CMCS/dECM Hydrogel and AS@PDA/ZIF-8 Composite Hydrogel

##### Mechanical Property Measurement

For tensile testing, the hydrogel samples were cast into dumbbell-shaped molds (70 × 4 × 1 mm) and allowed to equilibrate for 3 h. Uniaxial tensile tests were subsequently conducted at room temperature using an electronic universal testing machine equipped with a 200 N load cell, operating at a crosshead speed of 2 mm/min. The tensile stress–strain curves were recorded until sample fracture. For compressive testing, hydrogels of varying mass ratios were fabricated into cylindrical specimens (9 mm in diameter and 9 mm in height). Compressive tests were performed using the same testing apparatus at a compression rate of 2 mm/min, and the compressive stress–strain profiles were continuously recorded until structural failure. All mechanical tests were performed in triplicate for each experimental group to ensure reproducibility.

##### Porosity Test

The freeze-dried hydrogel sample was weighed and the initial weight was recorded as M_d_. After that, the sample was completely immersed in ethanol solution under vacuum. After 20 min, when the sample reached the absorption equilibrium, the residual liquid on the surface was wiped off, and the weight of the sample after the absorption saturation was weighed, which was recorded as M_S_. In order to ensure accuracy, the experimental results of each group were repeated three times and the average value was taken. The calculation formula of porosity (P_r_) is as follows:

P_r_ = (M_S_ − M_d_)/ρV × 100% (1)

In the formula, ρ is the density of ethanol and V is the volume of hydrogel.

##### Swelling Performance Test

The freeze-dried hydrogel samples were weighed, with the initial weight recorded as W_0_, and then immersed in PBS buffer with pH = 7.4. The samples were removed from the PBS buffer at different time points (2, 4, 6, 8, 10, 22, and 24 h). The water on the surface was wiped with filter paper and weighed, recorded as W_t_, and the samples continued to be weighed until the weight did not change. In order to ensure accuracy, the experimental results of each group were repeated three times and averaged. The swelling ratio (S_r_) of the hydrogel was calculated according to Formula (2):

S_r_ = (W_t_ − W_0_)/W_0_ × 100% (2)

##### Degradation Performance Test

The hydrogel samples were weighed and recorded as G_A,_ all immersed in 10 mL of simulated body fluid, and then placed in a shaker, 160 rpm, 37 °C. At different time points (3, 6, 9, 12, and 15 d), these samples were taken from the simulated body fluid and freeze-dried, and their weights were recorded as G_B_. In order to ensure accuracy, each group was averaged after three parallel tests. The degradation rate (D_r_) of the hydrogel was calculated according to Formula (3).

D_r_ = (G_A_ − G_B_)/G_A_ × 100% (3)

##### Electric-Conductivity Measure

The electrochemical properties of the gel were evaluated using an electrochemical workstation. The hydrogel was cut into circular pieces and clamped between two 1 mm thick stainless steel washers. The cross-sectional area S and thickness L of the hydrogel were measured. The impedance of the hydrogel was measured at an amplitude of 10 mV in the frequency range of 1 MHz to 100 MHz. Finally, the obtained hydrogel was subjected to electrochemical impedance spectroscopy (EIS), and the impedance R of the hydrogel was obtained by using the software fitting curve. The conductivity of the hydrogel was calculated using Formula (4).

σ = L/(R × S)(4)

##### Antibacterial Performance Test

In order to study the surface antibacterial activity of hydrogels, *Escherichia coli* (*E. coil*) and *Staphylococcus aureus* (*S. aureus*) were used for testing. The prepared hydrogel samples were sterilized via ultraviolet sterilization and placed in a 48-well plate. Subsequently, 100 μL of bacterial suspension (10^6^ CFU/mL) was dropped onto the surface of the hydrogel and cultured at 37 °C for 2 h. Subsequently, 1 mL of sterile PBS was added to each well to re-suspend the bacteria. The plate was coated with 40 μL suspension and incubated at 37 °C for 18 h to calculate the number of colonies on the agar plate [28]. The 100 μL bacterial suspension was dispersed in 1 mL PBS as a negative control. Each experiment was repeated three times. The antibacterial ratio of the hydrogel was calculated using Equation (5):

Antibacterial ratio (%) = (C_c_ − C_s_)/C_c_ × 100% (5)

C_c_ is the number of colonies in the control group, and C_s_ is the number of colonies in the hydrogel group.

##### Photothermal Performance Test

The photothermal ability of the hydrogel was evaluated using a near-infrared laser with a wavelength of 808 nm. Different hydrogel samples were irradiated at a density of 1.0 W/cm^2^ for 5 min, and temperature changes were recorded with a thermal imaging camera every 30 s. Subsequently, the temperature changes were measured with 1 W/cm^2^ and different samples to explore the influencing factors of temperature rise. In addition, in order to study the photothermal stability of the hydrogel, four cycles of NIR on/off cycle experiments were carried out.

#### 2.8.5. Biocompatibility Experiment of PAMB-CMCS/dECM Hydrogel

##### In Vitro Hemolysis Experiment

The fresh blood of SD rats was taken from the vacuum blood collection tube containing anticoagulant, washed, and diluted with PBS to obtain 2% red blood cell suspension. The 50 mg hydrogel sample was ground, added to a centrifuge tube, supplemented with 5 mL normal saline, and incubated in a 37 °C water bath for 30 min. Then, 5 mL 2% red blood cell suspension was added and incubated in a 37 °C water bath for 1 h. The mixture was centrifuged at 1000 r/min for 10 min, and the absorbance of the supernatant was measured at 545 nm using an ultraviolet–visible spectrophotometer, which was recorded as A_1_. At the same time, another two centrifuge tubes were taken, one of which was supplemented with 5 mL normal saline and the other was supplemented with 5 mL deionized water, and then 5 mL 2% red blood cell suspension was added and incubated with the sample in a 37 °C water bath for 1 h. The deionized water group was used as the positive control group (Pos.), and its absorbance was recorded as A_2_; the saline group was used as the negative control group (Neg.), and its absorbance was recorded as A_3_. Each group of samples was measured 3 times in parallel, and the average value was taken. The hemolysis rate (H_r_) of the hydrogel was calculated according to Formula (6):

H_r_ = (A_1_ − A_3_)/(A_2_ − A_3_) × 100% (6)

##### Cell Wound Scratch Assay

A total of 3 × 10^5^ fibroblasts were seeded in a 6-well plate and cultured with a pipette (200 μL) along the marker line at the bottom of each well. After washing with PBS buffer to remove free cells and dead cells, the sample extract was added and cultured for 24 h. Finally, the cell image was taken using an inverted microscope (Echo Revolve, San Diego, CA, USA), and the cell area was calculated using ImageJ software (1.53t, NIH, USA). The formula for calculating cell migration rate is as follows:

Mobility = A_0_ − A_1_/A_0_ × 100% (7)

A_0_ is the initial scratch area, and A_1_ is the scratch area that has not healed at the 24 h time point.

##### In Vivo Wound Healing Experiment of PAMB-CMCS/dECM Hydrogel

All animal experiments were conducted in strict accordance with institutional guidelines and were approved by the Experimental Animal Ethics Committee of Wuhan University of Technology. Adult male Sprague-Dawley (SD) rats weighing 180–210 g were utilized to evaluate the in vivo wound healing efficacy of the hydrogels. Following anesthesia via intraperitoneal injection of pentobarbital sodium, a full-thickness circular skin defect (approximately 10 mm in diameter) was created on the dorsal area of each rat. The wounds were randomly assigned to receive treatments with normal saline (control), the free drug (AS), or the composite hydrogel, followed by coverage with sterile medical gauze and fixation with tape. The dressings were replaced every 2 days, and macroscopic wound closure was observed and photographically documented over a 14-day period. On days 7 and 14 post-wounding, the regenerated wound tissues, along with the adjacent normal skin, were harvested, fixed in 4% paraformaldehyde overnight, embedded in paraffin, and sectioned. To histologically evaluate skin tissue regeneration and collagen deposition, the tissue sections were subjected to H&E and Masson’s trichrome staining.

## 3. Results and Discussion

### 3.1. Structural Characterization of Nanoparticles

**Figure 2:**
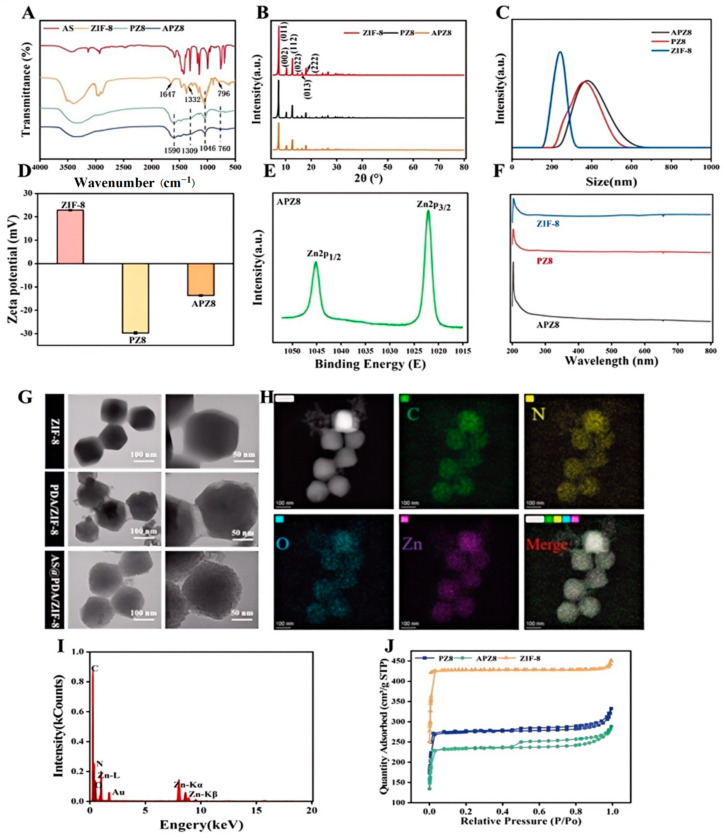
Microscopic morphology, crystalline structure, and surface chemical states of AS@PDA/ZIF-8 (APZ8) nanocomposites. (**A**) FTIR spectra, (**B**) XRD patterns, (**C**) DLS particle size distributions, and (**D**) Zeta potentials of ZIF-8, PZ8, and APZ8. (**E**) High-resolution XPS spectrum of Zn 2p for APZ8. (**F**) UV-vis absorption spectra of ZIF-8, PZ8, and APZ8. (**G**) TEM images of ZIF-8, PZ8, and APZ8. (**H**) HAADF-STEM image and corresponding EDS elemental mappings (C, N, O, Zn) of APZ8. (**I**) EDS spectrum of APZ8. (**J**) N_2_ adsorption−desorption isotherms of ZIF-8, PZ8, and APZ8.

The stepwise construction of APZ8 was first examined via FTIR and XRD analyses (Figure 2A,B). In the FTIR spectra, AS showed a broad O-H stretching band at 3200–3600 cm^−1^, C-H stretching vibrations at 2800–3000 cm^−1^, and C-O stretching signals at 1000–1100 cm^−1^. ZIF-8 showed imidazole-related characteristic peaks at 1332 and 796 cm^−1^, which proved that ZIF-8 was successfully prepared. The peak at 1647 cm^−1^ may be due to excessive ligand; centrifugal washing does not completely remove the free 2-methylimidazole (Figure S1). After PDA coating, these peaks were weakened, accompanied by the appearance of a broadband of about 3400 cm^−1^, indicating the successful introduction of PDA [29]. After AS loading, APZ8 retained the ZIF-8-related peak at 1332 cm^−1^ and showed an AS-associated C-O peak at 1046 cm^−1^. The broadened and shifted O-H band in APZ8 further suggests possible hydrogen-bonding interactions between AS and the PDA layer. XRD patterns (Figure 2B) showed that the characteristic diffraction peaks of ZIF-8 were largely preserved after PDA coating and AS loading, although the peak intensity slightly decreased, indicating that the ZIF-8 framework was maintained during modification and loading [30]. DLS and zeta potential measurements further supported the stepwise modification process (Figure 2C,D). The hydrodynamic diameter increased from approximately 220 nm for ZIF-8 to 380 nm for PZ8 and further to 400 nm for APZ8, consistent with PDA coating and subsequent AS loading [31]. Meanwhile, the zeta potential changed from +23.0 mV for ZIF-8 to –30.0 mV after PDA coating and then increased to –14.0 mV after AS loading, indicating surface charge regulation during the fabrication process. The monomodal size distribution of APZ8 suggests that no severe aggregation occurred after drug loading. XPS analysis showed two Zn 2p peaks at 1022.0 and 1045.1 eV, with a spin-orbit splitting of approximately 23.1 eV, indicating that the Zn coordination environment in ZIF-8 was largely retained after PDA coating and AS loading (Figure 2E). UV-Vis-NIR spectra showed that pristine ZIF-8 mainly absorbed in the UV region, whereas PZ8 and APZ8 exhibited broad absorption across the visible and NIR regions, which can be attributed to the PDA layer (Figure 2F). Notably, APZ8 maintained absorption near 800 nm, suggesting its potential for NIR-responsive behavior. TEM images showed that pristine ZIF-8 nanoparticles possessed a typical polyhedral morphology with sizes in the range of 50–200 nm (Figure 2G). After PDA coating and AS loading, the particles retained their overall morphology, while the surface became slightly rougher. EDS mapping confirmed the presence and distribution of C, N, O, and Zn elements in APZ8 (Figure 2H). N_2_ adsorption–desorption analysis showed Type I isotherms for the samples, while the adsorption volume decreased from approximately 425 cm^3^/g for ZIF-8 to 275 cm^3^/g for PZ8 and 230 cm^3^/g for APZ8 (Figure 2J), indicating reduced accessible porosity after PDA coating and AS loading. Overall, these results demonstrate the successful fabrication of AS-loaded PDA@ZIF-8 nanoparticles [32,33,34]. The ZIF-8 framework was largely preserved after surface modification and drug loading, while PDA coating contributed to surface charge reversal and visible-NIR absorption. The decreased adsorption capacity after AS loading further supports the occupation or partial blocking of ZIF-8 pores, providing a structural basis for subsequent release and NIR-responsive studies. HPLC analysis was performed to verify AS loading in the PDA-coated ZIF-8 nanocarriers [35]. As shown in Figure S2A–C, free AS displayed a sharp characteristic peak at approximately 11.2 min, whereas drug-free PZ8 showed no obvious signal at this retention time. In the APZ8 chromatogram, a corresponding AS peak appeared at 11.246 min, indicating the successful loading of AS into or onto the PZ8 nanocarriers. Drug-loading performance was further quantified using a standard calibration curve with good linearity (Figure S2D). As shown in Figure S2F, APZ8 exhibited a drug-loading capacity of 23.9 ± 1.5% and an encapsulation efficiency of 62.1 ± 1.8%. These results suggest that the PZ8 nanocarriers provided effective loading of AS. The in vitro release behavior of APZ8 was then evaluated in PBS at pH 7.4 and 37 °C (Figure S2E). APZ8 showed a relatively rapid initial release within the first 12 h, with approximately 15–18% cumulative release, followed by a slower release phase up to 72 h. The final cumulative release reached approximately 26%. This release profile indicates that APZ8 could provide sustained AS release under physiological conditions. Overall, these experimental data clearly indicate that we have successfully developed an APZ8 nanocarrier with high loading capacity and precise sustained release characteristics. Many literature sources on healing materials have pointed out that wound healing is a highly dynamic and complex physiological process regulated by multiple cells [36,37]. The drug release behavior of APZ8 in this study can perfectly match the long-term time span requirements of the wound from early anti-inflammatory to late microvascular regeneration, thus avoiding the drastic fluctuation of local drug concentration [38]. Therefore, the successful construction of the nano-delivery system not only strongly demonstrates the unique advantages of nanotechnology in the bioavailability of unstable bioactive factors but also serves as a core driving unit, paving the way for further anchoring it in a three-dimensional hydrogel network to achieve space–time controllable drug release [39,40].

### 3.2. Decellularized Extracellular Matrix Extraction Process and Immunogenicity Characterization

**Figure 3:**
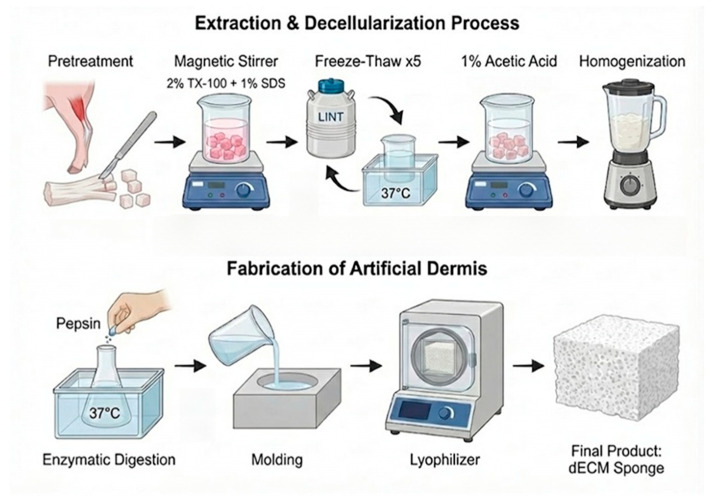
Process flow diagram of decellularization.

**Figure 4:**
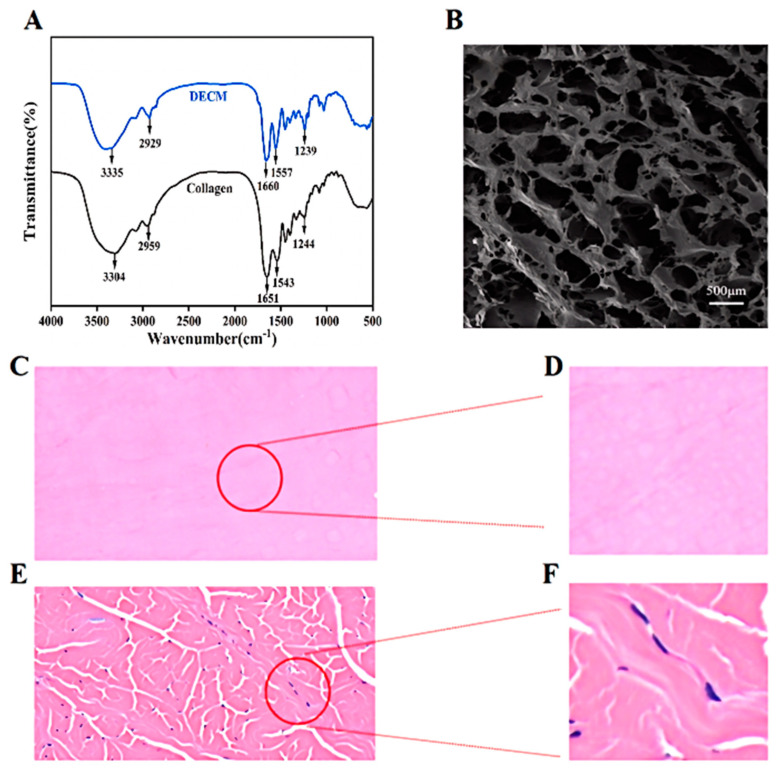
(**A**) Comparison of FT-IR spectra of dECM and natural collagen; (**B**) SEM images of dECM; (**C**–**F**) H&E staining analysis of tissue sections. (**E**,**F**) Untreated primary tissues showed a large number of blue-stained nuclei; the dECM prepared in (**C**,**D**) showed that the nucleus was completely cleared and the complete pink collagen fiber network was retained, which proved good acellular effect and low immunogenicity.

Recapitulating the biochemical composition and topological architecture of the native ECM is crucial for initiating the wound healing cascade. To validate the fabricated dECM as a viable regenerative scaffold (Figure 3 details the protocol), we systematically evaluated its immunocompatibility and physicochemical properties. Achieving true “immune inertness” to avert host rejection hinges on the thorough clearance of immunogenic nuclear components. Visual assessment via H&E staining confirmed this decellularization efficacy; whereas native tissue exhibited dense, blue-stained nuclear signals (Figure 4E,F), the dECM presented complete acellularity (Figure 4C,D). By preserving only the highly aligned, pink-stained collagen fibril network without residual nuclear fragments, this process effectively eliminates xenogeneic antigens, establishing the essential in vivo biosafety required for downstream applications [41,42,43]. Beyond mere biosafety, faithfully mimicking the native cellular microenvironment demands the maximal retention of biochemical cues. FT-IR spectroscopy (Figure 4A) confirmed that the dECM retained the characteristic fingerprint spectra of native collagen. Crucially, the absorption peaks corresponding to the protein secondary structure—Amide I (1660 cm^−1^ for C=O stretching) and Amide II (1557 cm^−1^ for N–H bending)—remained distinct and unshifted. Such structural integrity indicates that the mild decellularization protocol successfully protected the triple-helical conformation of collagen [44], thereby preserving intrinsic bioactive sites (e.g., RGD adhesion sequences) that intrinsically govern fibroblast adhesion, proliferation, and phenotypic maintenance. The micro-topological architecture directly dictates both mass exchange and cellular behavior. SEM imaging (Figure 4B) revealed a highly interconnected, three-dimensional porous network closely resembling native ECM. Rather than serving merely as a passive structural template, this biomimetic porosity provides the requisite physical space and topographical guidance for deep cellular infiltration, while simultaneously establishing a robust mass transport network. This dual-functioning architecture ensures the efficient drainage of wound exudates alongside the seamless permeation of oxygen and nutrients, ultimately fostering a microenvironment primed for robust vascularization and granulation tissue remodeling [45].

### 3.3. Synthesis Mechanism of PAMB-CMCS/dECM Conductive Hydrogel and Macroscopic and Microscopic Images

**Figure 5:**
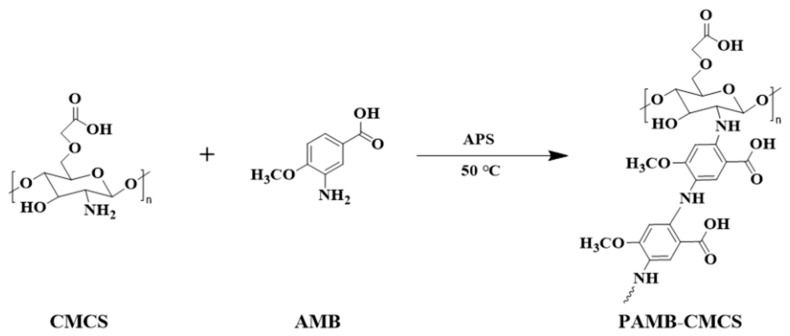
The schematic diagram of PAMB-CMCS synthesis mechanism.

**Figure 6:**
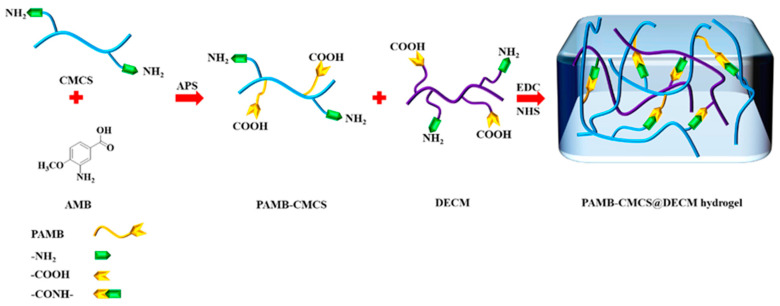
Preparation of PAMB-CMCS/dECM hydrogel.

**Figure 7:**
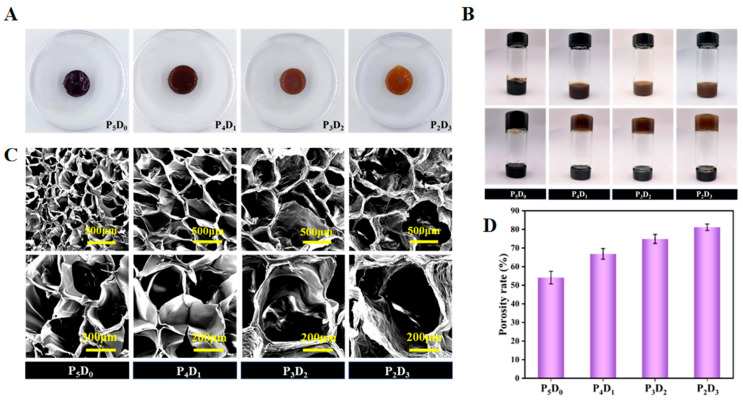
Macroscopic appearance, internal interconnected porous microstructures, and quantitative porosity of the composite hydrogels with varying formulation ratios. (**A**) Digital photographs displaying the gross morphology and optical distinctness of the hydrogels (P_5_D_0_, P_4_D_1_, P_3_D_2_, and P_2_D_3_) in the swollen state. (**B**) Cross-sectional SEM micrographs highlighting the multi-scale, highly interconnected 3D porous networks at different magnifications (scale bars: 500 μm, 300 μm, and 200 μm). (**C**) Optical images of the tube-inverting test confirming the successful sol–gel transition and shape-retention capability of the respective hydrogel formulations. (**D**) Quantitative evaluation of the hydrogel porosity rate (%) showing a tailored dependency on the component ratios (n = 3, mean ± SD).

By integrating the electroactive PAMB-CMCS matrix with the highly bioactive dECM at specific stoichiometric ratios, we engineered a series of biomimetic scaffolds that seamlessly couple electrophysiological cues with biochemical signals. The FT-IR spectra of hydrogels before and after modification of CMCS and after loading dECM are shown in Figure S3. Figure 5 and Figure 6 illustrate the synthesis mechanism of PAMB-CMCS and the subsequent fabrication of the composite hydrogels, respectively. Macroscopically (Figure 7A), increasing the dECM content from 0 to 60 wt% yielded a uniform color gradient, transitioning smoothly from deep black to light brown. This homogenous coloration visually substantiates the thorough, molecular-level integration of the components, devoid of any macroscopic phase separation. To assess their applicability for in situ filling, the sol–gel transition was evaluated via a vial inversion test (Figure 7B). Hydrogels across all compositions rapidly formed stable networks under physiological conditions, demonstrating excellent anti-gravity properties [46]. Such rapid gelation underscores their clinical translational potential for conformal adaptation within irregular wound beds. SEM (Figure 7C) revealed that while the pristine conductive hydrogel (P_5_D_0_) possessed a relatively dense network that might sterically hinder cellular infiltration [47], the incorporation of dECM profoundly modulated the structural porosity. As the dECM proportion increased (from P_4_D_1_ to P_2_D_3_), the average pore size of the hydrogel showed a clear increasing trend. As shown in the low-rate SEM image (500 μm), the P_5_D_0_ component exhibits a relatively dense honeycomb structure with a small pore size; when the proportion of dECM gradually increased, the pores expanded significantly, and a clear macro-macroporous network was formed when the P_2_D_3_ component was added. High-rate SEM images (200 μm) further confirmed that with the increase in dECM, the thickness of the pore wall and the space bridging structure were regulated, the boundary of the hole was clear, and no obvious network collapse occurred, indicating that the material maintained good three-dimensional structural integrity while expanding the pore size. The quantitative porosity test results (Figure 7D) are highly consistent with the evolution trend of microstructure observed via SEM. With the increase of the proportion of dECM components, the porosity of the hydrogel showed a monotonically increasing trend, and the specific value was significantly increased from 53.8% ± 3.5% of P_5_D_0_ to 81.2% ± 1.8% of P_2_D_3_. This phenomenon can be attributed to the physical and chemical mechanism: the introduction of the dECM component changes the crosslinking density of the precursor solution or the spatial arrangement of the macromolecular chains and reduces the spatial binding force of the hydrogel network, so that the ice crystals can fully grow into larger aggregates during the freezing process, and finally gives the material a higher proportion of space voids after freeze-drying.

### 3.4. Performance and Biological Characterisation of PAMB-CMCS/dECM Hydrogel

**Figure 8:**
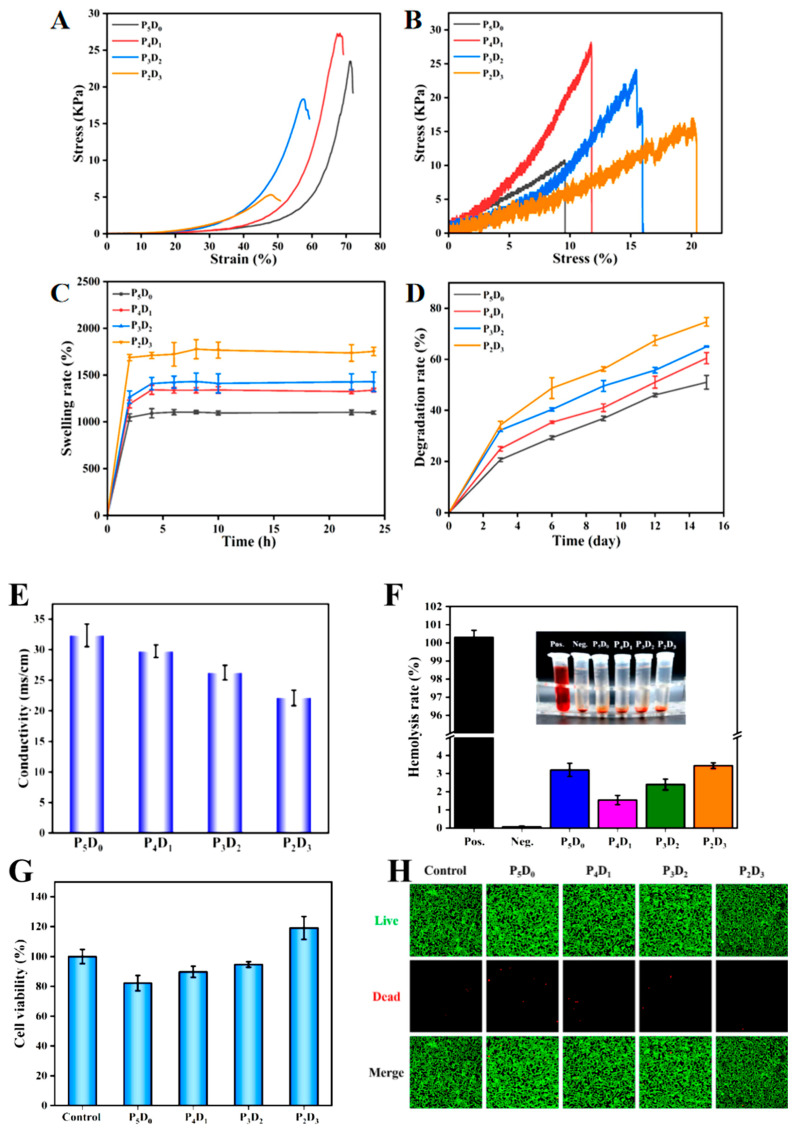
(**A**) Tensile stress–strain curve of hydrogel; (**B**) compressive stress–strain curve of hydrogel; (**C**) liquid absorption histograms of P_5_D_0_, P_4_D_1_, P_3_D_2_, and P_2_D_3_ hydrogels; (**D**) degradation rate statistics of P_5_D_0_, P_4_D_1_, P_3_D_2_, and P_2_D_3_ hydrogels within 15 days; (**E**) electrical conductivity histograms of P_5_D_0_, P_4_D_1_, P_3_D_2_, and P_2_D_3_ hydrogels; (**F**) hemolysis rate histogram and hemolysis experiment diagram of P_5_D_0_, P_4_D_1_, P_3_D_2_, and P_2_D_3_ hydrogels; (**G**) cell viability histograms of P_5_D_0_, P_4_D_1_, P_3_D_2_, and P_2_D_3_ hydrogels; (**H**) live/dead staining of P_5_D_0_, P_4_D_1_, P_3_D_2_, and P_2_D_3_ hydrogels.

The physicochemical and biological properties of PAMB-CMCS/dECM composite hydrogels were evaluated to screen an appropriate formulation for subsequent studies (Figure 8). As shown in Figure 8A,B, the incorporation of dECM affected both compressive and tensile properties. Among the tested formulations, P_4_D_1_ exhibited the highest compressive strength of 27.24 ± 1.9 kPa and tensile strength of 28.2 ± 2.8 kPa. In contrast, further increasing the dECM content reduced the mechanical strength, with P_2_D_3_ showing a compressive strength of 5.3 ± 1.1 kPa. Meanwhile, the elongation at break increased from 9.65 ± 0.33% in P_5_D_0_ to 20.36 ± 0.11% in P_2_D_3_, indicating that dECM improved the flexibility of the hydrogel network. Compared with the commercially available Kaltostat calcium sodium alginate wound dressing from Convatec in Figure S4, its mechanical properties are significantly improved, and its dual network structure provides a guarantee for the subsequent filling of nanoparticles. The swelling and degradation behaviors also showed dECM-dependent trends (Figure 8C,D). The swelling ratio increased with higher dECM content, and P_2_D_3_ reached the highest equilibrium swelling ratio of approximately 1750%. Similarly, the degradation rate increased as the dECM content increased, with P_2_D_3_ showing approximately 75% mass loss after 14 days. Compared with P_2_D_3_, P_4_D_1_ displayed more moderate swelling and degradation behavior, suggesting a better balance between water uptake and structural stability [48]. The conductivity of the hydrogels gradually decreased with increasing dECM content (Figure 8E). P_5_D_0_ showed the highest conductivity of 32.34 ± 1.86 mS/cm, while that of P_2_D_3_ decreased to 22.12 ± 1.27 mS/cm. This trend may be associated with the reduced proportion of the conductive PAMB-CMCS network after dECM incorporation. Nevertheless, all hydrogel groups retained measurable electrical conductivity. Hemolysis and cytocompatibility assays were further performed to evaluate the biosafety of the hydrogels (Figure 8F–H). All hydrogel groups showed hemolysis ratios below 5%, with P_4_D_1_ exhibiting the lowest value of 1.5 ± 0.26% and P_2_D_3_ showing 3.4 ± 0.15%, indicating acceptable hemocompatibility. CCK-8 results showed that fibroblast viability remained above 80% in all hydrogel groups after 24 h. Among them, P_2_D_3_ showed the highest cell viability of 112.44 ± 7.6%, while P_4_D_1_ maintained a viability of 89.76 ± 3.7%. Live/dead staining further showed abundant live cells and few dead cells on the hydrogel samples, supporting their cytocompatibility. Overall, P_4_D_1_ showed relatively high mechanical strength, moderate swelling and degradation behavior, retained conductivity, and acceptable hemocompatibility and cytocompatibility. Therefore, P_4_D_1_ was selected as the basic hydrogel matrix for subsequent functional modification and biological evaluation.

### 3.5. Characterisation of the Properties of P_4_D_1_, PZ8H, and APZ8H Hydrogels

**Figure 9:**
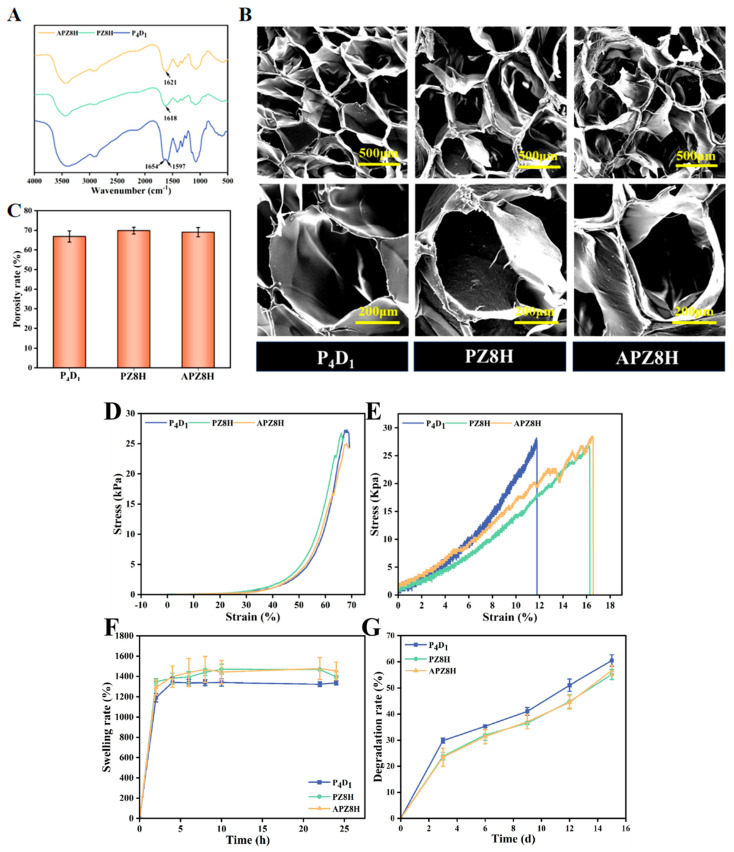
(**A**) Infrared spectra of P_4_D_1_, PZ8H, and APZ8H hydrogels; (**B**) SEM images of P_4_D_1_, PZ8H, and APZ8H composite hydrogels; (**C**) porosity of P_4_D_1_, PZ8H, and APZ8H hydrogels; (**D**) compressive stress−strain curves of P_4_D_1_, PZ8H, and APZ8H hydrogels; (**E**) tensile stress–strain curves of P_4_D_1_, PZ8H and APZ8H hydrogels; (**F**) swelling rate curves of P_4_D_1_, PZ8H, and APZ8H composite hydrogels; (**G**) degradation rate curves of P_4_D_1_, PZ8H, and APZ8H composite hydrogels.

APZ8 nanoparticles were incorporated into the optimized P_4_D_1_ hydrogel matrix to obtain the APZ8H composite hydrogel, and its structural and physicochemical properties were further evaluated (Figure 9). As shown in the FTIR spectra (Figure 9A), compared with P_4_D_1_, PZ8H and APZ8H showed changes in the fingerprint region. The characteristic peaks of P_4_D_1_ at 1654 and 1597 cm^−1^ shifted to a broader band around 1620 cm^−1^ after nanoparticle incorporation, suggesting possible interactions between the nanoparticles and the hydrogel network. SEM images showed that P_4_D_1_, PZ8H, and APZ8H all maintained interconnected porous structures (Figure 9B). No obvious pore collapse or severe nanoparticle aggregation was observed after the incorporation of PZ8 or APZ8. The porosity results also showed comparable porous characteristics among the three groups (Figure 9C), indicating that nanoparticle loading did not markedly disrupt the hydrogel architecture. The mechanical properties of the hydrogels were then assessed using compression and tensile tests (Figure 9D,E). The compressive strengths of P_4_D_1_, PZ8H, and APZ8H were 27.24 ± 1.9, 26.65 ± 1.7, and 24.91 ± 2.3 kPa, respectively, showing that APZ8H retained comparable compressive performance to the original hydrogel matrix. Tensile testing showed ultimate tensile stresses of 28.2 ± 2.8 kPa for P_4_D_1_, 26.73 ± 3.1 kPa for PZ8H, and 28.45 ± 2.3 kPa for APZ8H. In addition, APZ8H exhibited a higher elongation at break than P_4_D_1_, suggesting improved tensile deformability after APZ8 incorporation. The swelling profiles showed that all hydrogels rapidly absorbed water and reached equilibrium within several hours (Figure 9F). The equilibrium swelling ratios of P_4_D_1_, PZ8H, and APZ8H were 1341 ± 47%, 1466 ± 10%, and 1477 ± 11%, respectively. The slightly higher swelling ratios of PZ8H and APZ8H may be related to the introduction of hydrophilic nanoparticle components. In vitro degradation tests showed gradual mass loss in all groups over 15 days (Figure 9G). At day 15, the degradation rates of P_4_D_1_, PZ8H, and APZ8H were 60.47 ± 2.19%, 55.12 ± 1.96%, and 56.63 ± 1.93%, respectively. Compared with P_4_D_1_, the nanoparticle-containing hydrogels showed slightly slower degradation. Overall, APZ8 incorporation did not markedly damage the porous structure or mechanical integrity of the P_4_D_1_ hydrogel. APZ8H maintained suitable swelling behavior, moderate degradation, and comparable mechanical performance, supporting its use as the composite hydrogel formulation for subsequent biological evaluation [49].

### 3.6. Photothermal and Antioxidant Performance Tests of P_4_D_1_, PZ8H, and APZ8H Hydrogels

**Figure 10:**
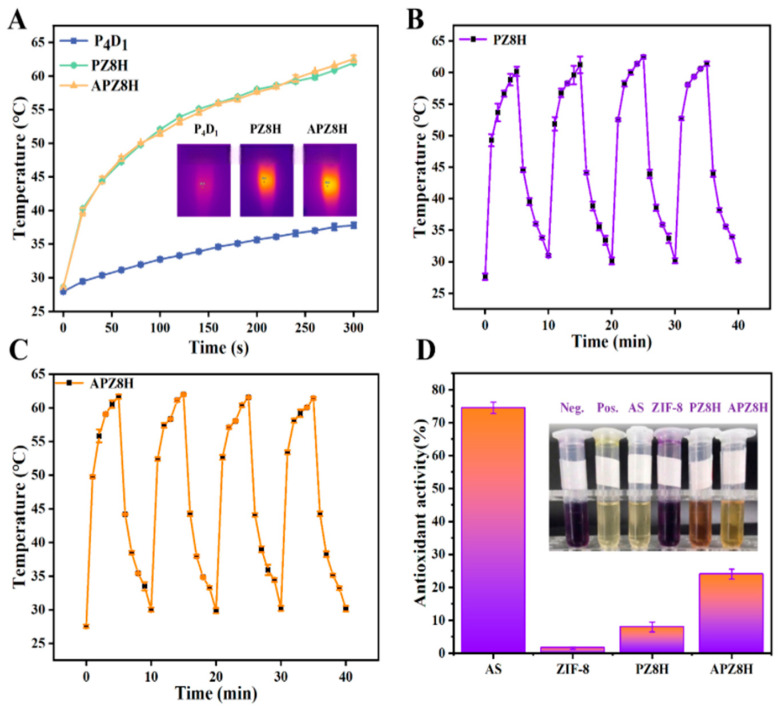
(**A**) The heating curves of P_4_D_1_, PZ8H, and APZ8H composite hydrogels under near-infrared laser irradiation at 808 nm, 1.0 W/cm^2^ for 5 min. (**B**,**C**) PZ8H and APZ8H temperature change curves in four laser switching cycles. (**D**) Macroscopic diagram and antioxidant activity histogram of DPPH free radical scavenging experiment of AS, ZIF-8, PZ8H, and APZ8H composite hydrogels.

The photothermal properties of hydrogels were evaluated under 808 nm, 1 W/cm^2^ near-infrared light irradiation (Figure 10A–D). As shown in Figure 10A, the P_4_D_1_ hydrogel exhibited only a slight temperature increase, reaching approximately 37.8 °C after 300 s. In contrast, PZ8H and APZ8H showed rapid temperature elevation, reaching around 62 °C within the same irradiation period. The corresponding infrared thermal images further confirmed that the temperatures of PZ8H and APZ8H exceeded 60 °C after 300 s, whereas P_4_D_1_ remained near physiological temperature. These results indicate that the PDA-coated nanoparticles endowed the hydrogels with effective NIR-responsive photothermal behavior [50]. The photothermal cycling curves showed that both PZ8H and APZ8H maintained similar heating and cooling profiles over four repeated laser on/off cycles (Figure 10B,C). No obvious decrease in the maximum temperature was observed, suggesting good photothermal stability after repeated irradiation. The antioxidant activity of the material was further evaluated using the DPPH method (Figure 10D). Compared with the negative control DPPH group, the supernatant of AS, PZ8H, and APZ8H showed obvious color fading, indicating that they had free radical scavenging activity. From the quantitative point of view, the free radical scavenging rate of AS is close to 80%, which has strong antioxidant properties. The ZIF-8 clearance rate of the carrier was close to 1%, and there was almost no antioxidant activity. The free radical scavenging rate decreased to about 25% after the AS was loaded with ZIF-8 and incorporated into the hydrogel system, which was because the drug-loading system formed a sustained release effect. In summary, APZ8H has both near-infrared responsive photothermal behavior and free radical scavenging ability, which may be beneficial to regulate the oxidative wound microenvironment.

### 3.7. Antibacterial Performance Testing of PZ8H and APZ8H Hydrogels

**Figure 11:**
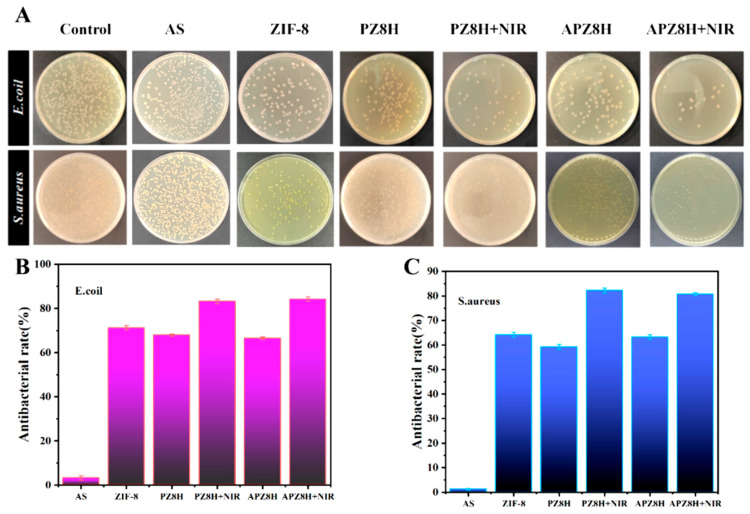
Near-infrared (NIR)-augmented in vitro antibacterial efficacy and quantitative colony-formation analysis of the APZ8H hydrogel system. (**A**) Representative digital photographs of re-cultivated agar plates showing the survival of *Escherichia coli* (*E. coli*) and *Staphylococcus aureus* (*S. aureus*) colonies after incubation with different formulations (control, AS, ZIF-8, PZ8H, PZ8H + NIR, APZ8H, and APZ8H + NIR) for 24 h. Corresponding quantitative antibacterial rates (%) calculated using the colony counting method against (**B**) *E. coli* and (**C**) *S. aureus*, highlighting the synergistic chemo-photothermal/photodynamic bactericidal action (n = 3, mean ± SD).

Bacterial infection and subsequent persistent inflammation are the main factors driving wound chronicity. Figure 11 shows the representative agar plate photos (Figure 11A) of *E. coli* and *S. aureus* incubated with various materials and the corresponding quantitative antibacterial efficiency against *E. coli* (Figure 11B) and *S. aureus* (Figure 11C). Macroscopic observation from the standard plate count test (Figure 11A) showed that the number of colonies in the ZIF-8 group was significantly lower than that in the AS group compared with the dense bacterial colonies in the control group. The number of viable colonies in the PZ8H group (only loaded with ZIF-8) was also significantly reduced. Quantitative analysis (Figure 11B,C) indicated that the antibacterial efficiency of the PZ8H hydrogel against *E. coli* and *S. aureus* reached 70.31 ± 1.6% and 60.61 ± 2.1%, respectively. This intrinsic antibacterial activity is primarily attributed to the controlled degradation of the ZIF-8 nanoparticles and the subsequent sustained release of zinc ions (Zn^2+^ within the mildly acidic microenvironment induced by bacterial metabolism. The liberated Zn^2+^ disrupts the bacterial membrane potential via electrostatic interactions and subsequently translocates intracellularly to interfere with essential enzymatic systems [51]. NIR irradiation, localized hyperthermia generated via the photothermal effect, induces the denaturation of bacterial membrane proteins and causes irreversible physical damage to the cell walls. As evidenced in Figure 11A, the agar plates of both the PZ8H + NIR and APZ8H + NIR groups displayed a near-complete eradication of visible colonies. Specifically, the bactericidal rates of the PZ8H + NIR group against *E. coli* and *S. aureus* increased to 82.75 ± 1.75% and 82.07 ± 2.61%, respectively; similarly, the APZ8H + NIR group achieved killing efficacies of 80.57 ± 2.26% and 78.00 ± 2.23% against the two respective strains. This enhanced bactericidal performance is attributed to a heat-assisted permeabilization mechanism. Localized hyperthermia not only exerts direct thermal cytotoxicity but also significantly increases the permeability of bacterial cell walls and membranes. This compromised barrier integrity accelerates the intracellular influx of Zn^2+^ and therapeutic molecules, resulting in highly efficacious synergistic sterilization.

### 3.8. Biocompatibility and Scratch Tests of PZ8H and APZ8H Hydrogels

**Figure 12:**
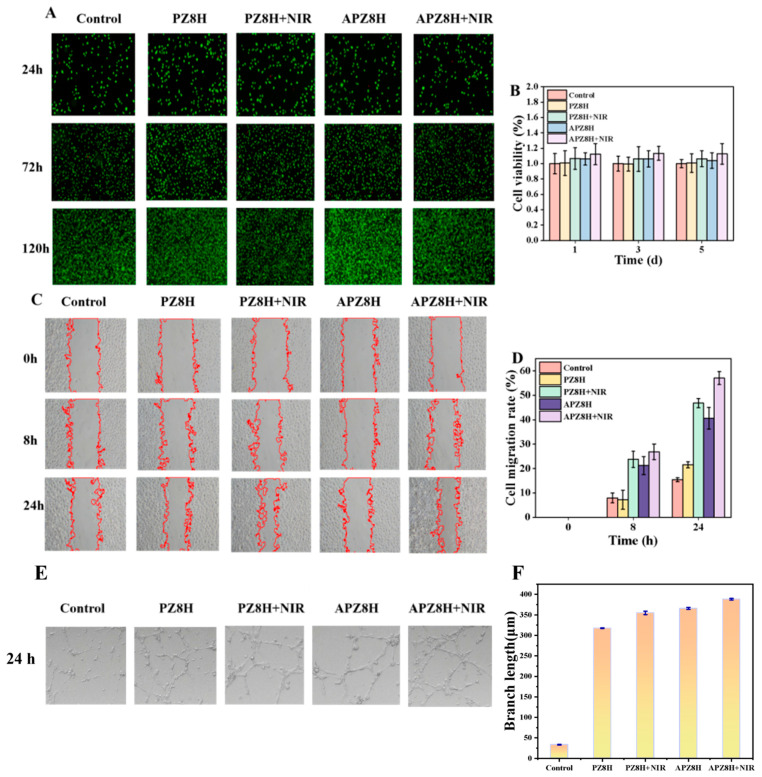
(**A**) Cell viability and death staining after 1, 3, and 5 days of co-culture of composite hydrogel and control group cells (n = 3). (**B**) Cell viability histogram. (**C**) Cell scratch macrograph of composite hydrogel and control group (n = 3). (**D**) Cell migration rate histogram. (**E**) Representative micrographs of HUVEC angiogenesis after 24 h of hydrogel extract and combined NIR action (n = 3). (**F**) Quantitative analysis of reticular tube length in HUVECs.

**Figure 13:**
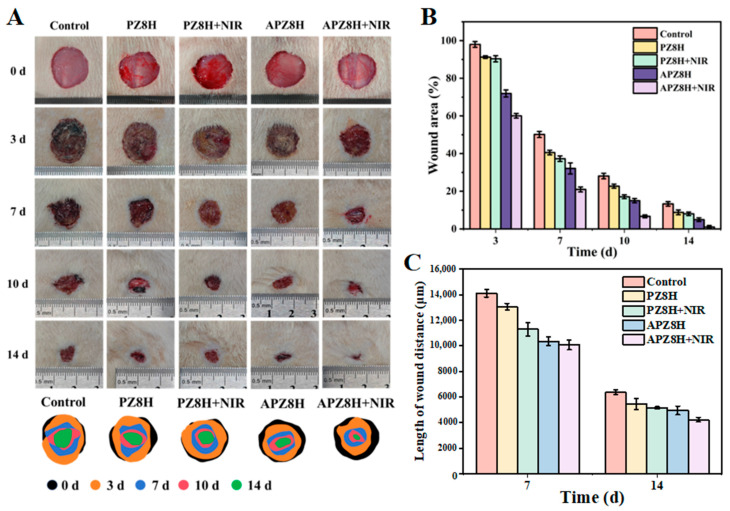
(**A**) Fourteen days of wound healing and wound edge tracing of the composite hydrogel and control group (n = 5); (**B**) percentage of wound unhealed area of the composite hydrogel and control group histogram; (**C**) wound length statistics.

The in vitro cytocompatibility of the hydrogels was evaluated using L929 fibroblasts through live/dead staining and CCK-8 assays (Figure 12A,B). As shown in Figure 13A, cells in all groups, including the control, PZ8H, PZ8H + NIR, APZ8H, and APZ8H + NIR, displayed predominantly green fluorescence with only a few red-stained dead cells during the 5-day culture period. The fluorescence density increased over time, indicating continuous cell growth. These results suggest that the hydrogels and the applied NIR treatment did not induce obvious cytotoxicity under the tested conditions [52]. The CCK-8 results further supported the live/dead staining observations (Figure 12B). Cell viability was maintained in all hydrogel groups from day 1 to day 5. Compared with PZ8H, APZ8H showed improved cell viability, and the APZ8H + NIR group exhibited the highest viability among the tested groups, suggesting that AS loading and NIR-responsive treatment may contribute to a more favorable cellular response. Fibroblast migration was further assessed using a scratch assay (Figure 12C,D). At 0 h, the scratch gaps were comparable among all groups. After 24 h, all groups showed wound-gap narrowing, while the APZ8H + NIR group exhibited the most pronounced closure. Quantitatively, the migration rate increased from 15.4 ± 0.83% in the control group to 21.52 ± 1.22% in the PZ8H group and 40.66 ± 0.44% in the APZ8H group. The APZ8H + NIR group showed the highest migration rate of 57.05 ± 2.65%. Overall, these results indicate that APZ8H maintained good cytocompatibility and promoted L929 fibroblast migration, particularly under NIR irradiation. This provides in vitro evidence supporting its potential role in enhancing wound-related cellular responses [53]. From Figure 12E, we can see the microscopic morphology of the capillary-like network structure formed by each group of cells. Compared with control, the cell connectivity and network integrity of PZ8H, PZ8H + NIR, APZ8H, and APZ8H + NIR groups increased in turn. Among them, the cells in the control group were mostly dispersed or incompletely connected; the tubular network formed in the APZ8H + NIR group was the most dense and complete. The quantitative analysis of Figure 12F showed that the branch length of the control group was the lowest, about 35 μm. The branch length of the PZ8H group increased significantly, about 320 μm. The branch length of the PZ8H + NIR group (355 μm) and APZ8H group (365 μm) was further increased compared to that of the PZ8H group. The branch length of the APZ8H + NIR group reached the maximum value of about 390 μm. Both PZ8H and APZ8H can effectively promote endothelial cell tube formation, and the promotion effect of APZ8H is better than that of PZ8H. The introduction of NIR irradiation can further enhance the tube-forming effect of both, and the combined treatment of APZ8H + NIR showed the strongest ability to promote angiogenesis.

### 3.9. In Vivo Wound Healing

The in vivo wound repair performance of the hydrogels was evaluated using a rat full-thickness skin defect model (Figure 13). As shown in Figure 13A, all groups exhibited comparable initial wound areas on day 1. During the healing period, the hydrogel-treated groups showed faster wound contraction than the control group, with the most obvious improvement observed in the APZ8H + NIR group. By day 14, the APZ8H + NIR group displayed nearly complete macroscopic wound closure, while residual wound areas were still visible in the control and other treatment groups. Quantitative analysis further confirmed these observations (Figure 13B). On day 3, the residual wound area of the APZ8H + NIR group decreased to 60.04 ± 1.23%, which was lower than that of the control group, 97.98 ± 1.54%. This trend continued throughout the observation period. By day 10, the residual wound area in the APZ8H + NIR group was 6.65 ± 0.77%, compared with 28.09 ± 1.46% in the control group. At day 14, APZ8H + NIR showed the smallest residual wound area, 0.98 ± 0.77%, followed by APZ8H, 4.92 ± 0.96%, PZ8H + NIR, 7.98 ± 0.94%, PZ8H, 8.76 ± 1.27%, and the control group, 13.29 ± 1.22%. The wound length analysis showed a similar tendency (Figure 13C). At day 14, the APZ8H + NIR group exhibited the shortest residual wound length of 4219.83 ± 176.73 μm, whereas the control group remained at 6353.73 ± 173.25 μm. These results indicate that APZ8H combined with NIR irradiation effectively promoted wound closure in vivo [54]. Overall, the APZ8H + NIR treatment showed the best wound healing performance among the tested groups, as evidenced by the smallest residual wound area and wound length. This improvement may be related to the combined contribution of the hydrogel matrix, APZ8 incorporation, and NIR-responsive behavior, although the detailed mechanism requires further histological and molecular evidence.

### 3.10. H&E and Masson Experiments of Hydrogel-Healed Wounds

**Figure 14:**
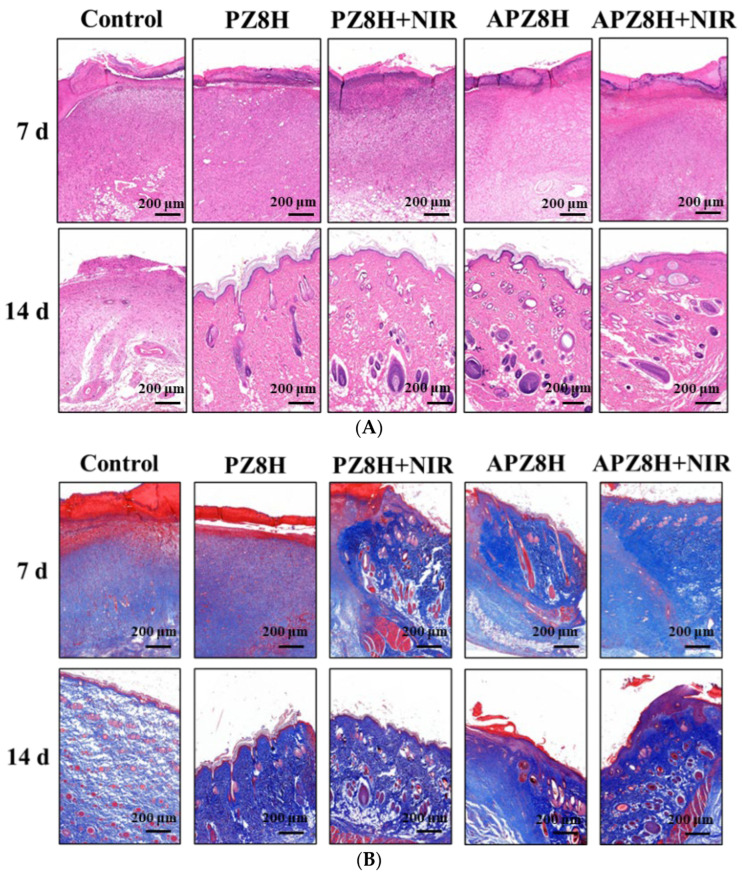
(**A**) HE staining of wound tissue sections of the composite hydrogel and control group on day 7 and day 14. (**B**) Masson staining of wound tissue sections of the composite hydrogel and control group on day 7 and day 14.

Histological evaluation was performed using H&E and Masson trichrome staining to further assess wound repair quality (Figure 14). As shown by H&E staining, the control group exhibited incomplete epidermal coverage and relatively disorganized dermal tissue at day 7. In contrast, the hydrogel-treated groups showed improved tissue coverage to varying degrees, with APZ8H + NIR displaying a more continuous epithelial layer and reduced wound gap. By day 14, tissue repair was improved in all groups. Compared with the control group, the APZ8H and APZ8H + NIR groups exhibited more complete epidermal reconstruction and denser dermal tissue. In particular, APZ8H + NIR showed a relatively continuous epidermis and more evident skin appendage-like structures, indicating improved histological repair compared with the untreated control [55,56]. Masson trichrome staining was used to evaluate collagen deposition and extracellular matrix remodeling [57,58,59]. At day 7, collagen deposition was limited in the control group, while stronger blue staining was observed in the hydrogel-treated groups. By day 14, collagen deposition increased in all groups, with APZ8H and APZ8H + NIR showing more abundant and compact collagen staining than the control group. Among them, APZ8H + NIR exhibited the most pronounced collagen deposition, consistent with its faster macroscopic wound closure. Overall, these histological results suggest that APZ8H, especially combined with NIR irradiation, promoted epidermal reconstruction, dermal tissue formation, and collagen deposition during wound repair.

## 4. Conclusions

In summary, we have successfully engineered an APZ8H nanocomposite hydrogel, a multifunctional platform designed to overcome the complex challenges of hostile wound microenvironments. By seamlessly integrating an electroconductive dECM-based matrix with AS-loaded ZIF-8 nanoparticles, this biomaterial achieves a profound synergy between biophysical guidance and targeted pharmacological therapy. Biomechanically, the hydrogel exhibits optimized compressive (24.91 ± 2.3 kPa) and tensile (28.45 ± 2.3 kPa) properties, ensuring conformal and stable interfacing with dynamic wound beds. Crucially, its tissue-mimicking electrical conductivity (29.84 ± 2.03 mS/cm) effectively bridges defect gaps to restore endogenous electrophysiological signaling networks. Beyond structural and biophysical support, the engineered dressing delivers robust antimicrobial efficacy—achieving bactericidal rates of approximately 90% and 98% against *Staphylococcus aureus* and *Escherichia coli*, respectively. This is complemented by an intrinsic antioxidant capacity and controlled biodegradability, which collectively facilitate dynamic tissue integration. Rigorous in vitro assessments confirm the material’s exceptional biosafety, evidenced by strict hemocompatibility (hemolysis < 5%) and sustained cytocompatibility (viability > 80%). Ultimately, comprehensive in vivo evaluations using a Sprague Dawley (SD) rat full-thickness skin defect model validate that the APZ8H intervention comprehensively remodels the wound microenvironment, significantly accelerating the healing cascade and driving high-quality, functional tissue regeneration. Collectively, this coupled biophysical–pharmacological strategy presents a highly promising paradigm for advanced clinical wound management.

## Data Availability

Data will be made available upon request.

## Supplementary Materials

The following supporting information can be downloaded at: https://www.mdpi.com/article/10.3390/pharmaceutics18060726/s1, Figure S1: Infrared spectra of 2-methylimidazole; Figure S2: The representative HPLC chromatograms of (A–C) astragaloside (AS), polydopamine-coated ZIF-8 (PZ8) and drug-loaded complex (APZ8). (D) Construction of standard curve of astragaloside IV at different concentrations (>0.99). (E) The in vitro cumulative release curve of APZ8 under physiological conditions (PBS, pH 7.4, 37 °C) over time. Data points represent mean ± standard deviation (n = 3). (F) The drug loading (DL) and encapsulation efficiency (LR) histogram of APZ8; Figure S3: FT-IR of CMCS before and after modification. (b) FT-IR diagram of composite hydrogel; Figure S4: (a) Convatec ’s Kaltostat calcium sodium alginate wound dressing tensile stress-strain curve. (b) Kaltostat calcium sodium alginate wound dressing compressive stress-strain curve. Figure S5: Conductive hydrogel lights up LED physical map.
