# Supplementary material for: Astragaloside IV-Loaded Polydopamine/Zeolitic Imidazolate Framework-8 Nanoparticles Embedded in Conductive Decellularized Extracellular Matrix-Modified Hydrogels for Wound Healing
Source: Pharmaceutics. 2026 Jun 12;18(6):726. doi: 10.3390/pharmaceutics18060726 (PMC13306435; doi:10.3390/pharmaceutics18060726)
Supplement: Supplementary file 1 [file pharmaceutics-18-00726-s001.zip › pharmaceutics-4354473-supplementary.pdf]

Supporting Information

Astragaloside IV-Loaded Polydopamine/Zeolitic Imidazolate Framework-8 Nanoparticles Embedded in Conductive Decellularized Extracellular Matrix-Modified Hydrogels for Wound Healing

S1. Experimental Section

S1.1 Antioxidant ability of hydrogels using chemical assay kits

S1.1.1 DPPH Free Radical Scavenging Experiment

The working solution and positive control were prepared according to the kit instructions. For each hydrogel group, 20 mg of ffreeze-dried hydrogel was weighed, and 1 mL of extract solution was added for the extraction of antioxidants. The extraction process was carried out for 30 min. Following the instructions, the absorbance of the negative control, positive control, blank, and experimental samples was measured at 515 nm using a UV spectrophotometer. The radical scavenging activity was calculated based on the formula provided in the kit instructions.

S1.1.2 Total Antioxidant Capacity of hydrogels

The standard curve solution was prepared and measured following the kit instructions. For each hydrogel group, 5 mg of freeze-dried hydrogel was weighed, and 1 mL of extract solution was added for antioxidant extraction. The subsequent steps were performed according to the manufacturer's instructions. The absorbance was measured at 593 nm using a spectrophotometer. Antioxidant activity was then calculated based on the provided formula in the kit instructions.

S1.1.3 *In vitro* cytotoxicity assay

Three 96-well plates were prepared for cell inoculation, and cell inventories of hydrogel at 24 h, 48 h, and 72 h were measured using the CCK-8 Cell Proliferation and Cytotoxicity Assay Kit (Solarbio, CA1210). L929 cells were cultured in DMEM supplemented with 10%FBS and 1% penicillin-streptomycin. The cells were incubated at 37°C in a 5% CO_2 humidified atmosphere. The hydrogels were sterilized under UV light for 12 h. To prepare the hydrogel extracts, hydrogels from each group were incubated in complete culture medium at a concentration of 2 mg/mL at 4°C for 48 h. After the extraction period, the extracts were filtered using a sterile 0.22 μm filter membrane to remove any particulate matter. The filtered extracts were then stored at 4°C for future use. L929 cells were seeded into 96-well plates at a density of 1×10^4 cells per well and incubated for 24 h to allow cell attachment. After the initial 24-h incubation, the culture medium was replaced with the hydrogel extract solution. For the positive control group , cells were treated with fresh culture medium, and for the negative control group, cells were treated with10% DMSO. A plate of cells was removed at 24 h, 48 h and 72 h, respectively, and detected with CCK-8 detection kit. For detection methods, refer to the kit instructions. Cell survival was calculated according to the following formula:

$$L929\ Cell\ Viability = \frac{OD_{sample} - OD_{negative}}{OD_{positive} - OD_{negative}} \times 100\%$$

Red-Green Staining of Live and Dead L929 Cells: The L929 cells cultured at 72 h were stained using the Calcein/PI Live/Dead Viability/Cytotoxicity Assay Kit (Beyotime, C2015S). For detection methods, refer to the kit instructions.

S1.1.4 In vitro angiogenesis

HUVECs were seeded on the surface of the 96-well plate at a density of 1 × 105 cells / well, and the extract obtained by soaking the hydrogel in the medium for 1 day was used for the above cell culture. After 24 hours of incubation, the medium was removed and the cells were fixed with 4 % paraformaldehyde. The formation of lumen was observed under a microscope. The tube length was measured and calculated using Image J software.

S1.2 Results

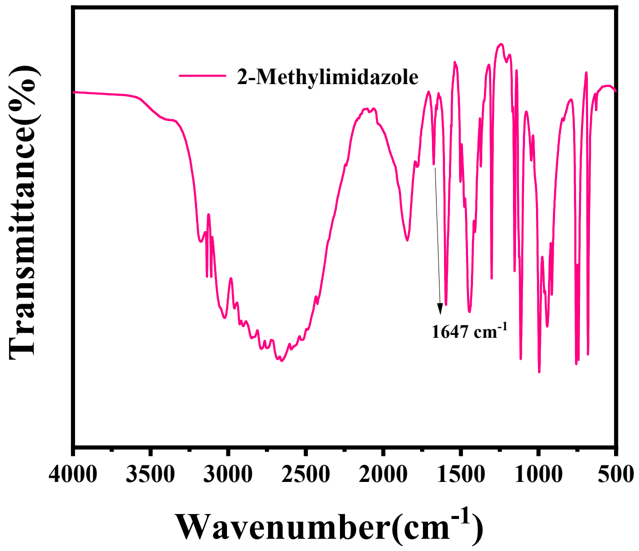

Figure S1. Infrared spectra of 2-methylimidazole

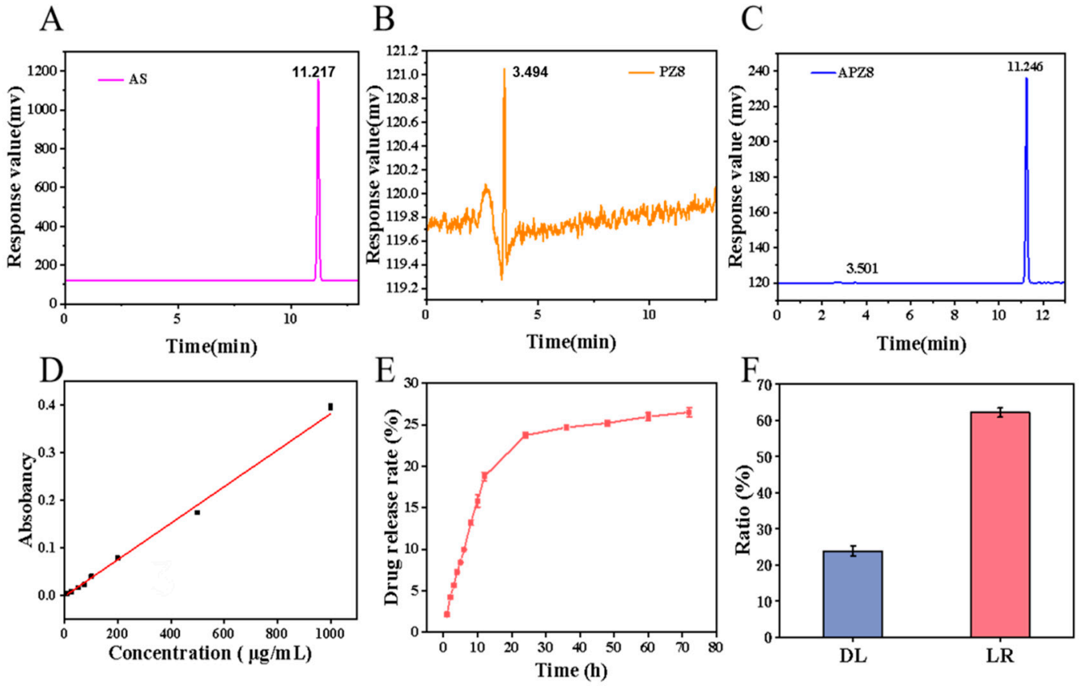

Figure S2. The representative HPLC chromatograms of (A-C) astragaloside (AS), polydopamine-coated ZIF-8 (PZ8) and drug-loaded complex (APZ8). (D) Construction of standard curve of astragaloside IV at different concentrations (> 0.99). (E) The in vitro cumulative release curve of APZ8 under physiological conditions (PBS, pH 7.4, 37 °C) over time. Data points represent mean ± standard deviation (n=3). (F) The drug loading (DL) and encapsulation efficiency (LR) histogram of APZ8.

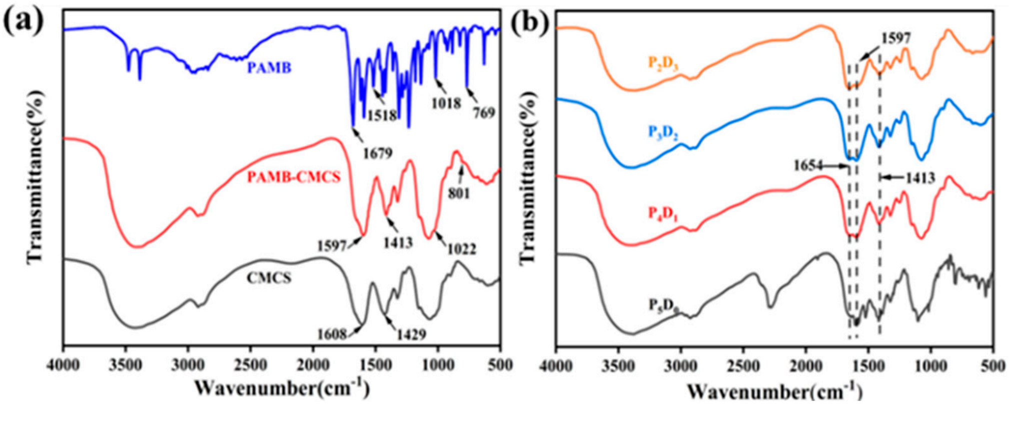

Figure S3. (a) FT-IR of CMCS before and after modification. (b)FT-IR diagram of composite hydrogel .

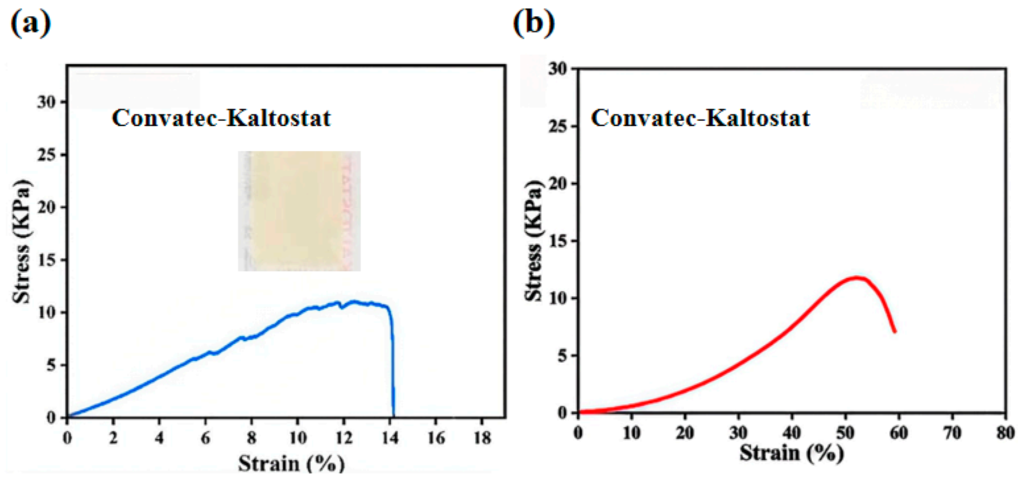

Figure S4. (a) Convatec 's Kaltostat calcium sodium alginate wound dressing tensile stress-strain curve (b) Kaltostat calcium sodium alginate wound dressing compressive stress-strain curve

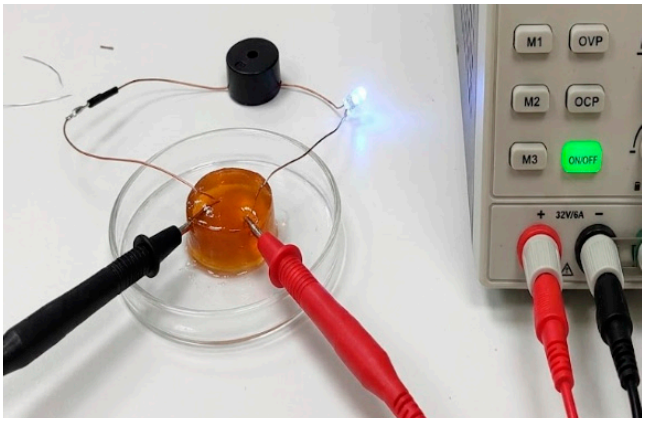

Figure S5. Conductive hydrogel lights up LED physical map
